# Downregulation of the Astroglial Connexin Expression and Neurodegeneration after Pilocarpine-Induced Status Epilepticus

**DOI:** 10.3390/ijms24010023

**Published:** 2022-12-20

**Authors:** Anna Andrioli, Paolo Francesco Fabene, Giuseppa Mudò, Vincenza Barresi, Valentina Di Liberto, Monica Frinchi, Marina Bentivoglio, Daniele Filippo Condorelli

**Affiliations:** 1 Department of Neuroscience, Biomedicine and Movement Sciences, University of Verona, 37134 Verona, Italy; 2 Verona Unit, National Institute of Neuroscience (INN), 37129 Verona, Italy; 3 Department of Biomedicine, Neuroscience and Advanced Diagnostic (BiND), University of Palermo, 90133 Palermo, Italy; 4 Unit of Medical Biochemistry, Department of Biomedical and Biotechnological Sciences, University of Catania, 95123 Catania, Italy

**Keywords:** gap junctions, electrical synapses, neurodegeneration, neuroinflammation, astrocytes, epilepsy

## Abstract

Astrocytic networks and gap junctional communication mediated by connexins (Cxs) have been repeatedly implicated in seizures, epileptogenesis, and epilepsy. However, the effect of seizures on Cx expression is controversial. The present study focused on the response of Cxs to status epilepticus (SE), which is in turn an epileptogenic insult. The expression of neuronal Cx36 and astrocytic Cx30 and Cx43 mRNAs was investigated in the brain of rats in the first day after pilocarpine-induced SE. In situ hybridization revealed a progressive decrease in Cx43 and Cx30 mRNA levels, significantly marked 24 h after SE onset in neocortical areas and the hippocampus, and in most thalamic domains, whereas Cx36 mRNA did not exhibit obvious changes. Regional evaluation with quantitative real-time-RT-PCR confirmed Cx43 and Cx30 mRNA downregulation 24 h after SE, when ongoing neuronal cell death was found in the same brain regions. Immunolabeling showed at the same time point marked a decrease in Cx43, microglia activation, and interleukin-1β induction in some microglial cells. The data showed a transient downregulation of astroglial Cxs in the cortical and thalamic areas in which SE triggers neurodegenerative events in concomitance with microglia activation and cytokine expression. This could potentially represent a protective response of neuroglial networks to SE-induced acute damage.

## 1. Introduction

Gap junctions are specialized membrane regions composed of aggregates of transmembrane channels that connect the cytoplasm of adjacent cells directly, allowing the for the intercellular movement of ions, metabolites, and second messengers [1,2,3]. Each intercellular channel is formed by two hemichannels, or connexons, formed by the hexameric assembly of subunit proteins, the connexins (Cxs). Several members of the large multigene Cx family are expressed in the brain; among them, Cx36 is expressed in neurons, mainly in GABAergic interneurons [1,4,5], while Cx43 and Cx30 are the main Cxs expressed in astrocytes [6,7,8].

It has been proposed that direct electrotonic interneuronal communication via gap junctions, in combination with synaptic and ionic mechanisms, could contribute to the generation or maintenance of seizures [9,10,11,12]. In humans, gap junctional coupling has been implicated in mesial temporal lobe epilepsy [13,14,15,16] and other seizure types [15,17,18,19]. Moreover, the altered expression of brain Cxs has been reported in epilepsy in in vivo and in vitro models, as well as in human epileptic brain tissue [16,20,21]. Gap junctional blockers have been found to suppress epileptic activity in vitro [22,23,24,25] and in vivo [25,26,27,28,29,30], as well as to have an excitatory effect in the isolated cerebral rat cortex [31] and to exacerbate glutamate toxic insult and cell mortality [21,32,33]. These findings are, however, controversial [12,20,32].

In particular, contradictory findings have been reported regarding the acute response of astrocytic Cxs to prolonged seizures. Previous studies have reported an early and transient upregulation of *Cx30* mRNA in several brain regions in response to kainate-induced seizures in rats, with the expression of this transcript in neurons undergoing apoptosis [34]. In the hippocampus, a differential response of Cx43 was found in different layers of the hippocampal CA3 field 2 h after the onset of pilocarpine-induced status epilepticus (SE) in rats [35], while the expression of astrocytic Cx43 was found to be unchanged [36] or increased [37] 24 h after SE in the same paradigm in the mouse hippocampus. The controversial results regarding Cxs alteration during the acute, silent, and chronic phases in the pilocarpine model of temporal lobe epilepsy (TLE) are reported in Table 1.

Unremitting seizures that configure SE not only represent one of the most intense in vivo activations of brain cells, but also cause neurodegenerative phenomena and other cell changes that subserve epileptogenesis [41]. Thus, SE provides a paradigm to study the responses of brain Cxs in vivo, in order to consider the pathologically elevated neuronal activity leading to acute and delayed neuronal cell death, with permanent and epileptogenic brain changes.

On this basis, the objective of the present study was to verify the neuronal and astrocytic Cxs mRNA and protein expression, in parallel with cell death, the glial response, and cytokines expression during the acute period (3 to 24 h) after SE in an animal model of prolonged seizures configuring SE, obtained by pilocarpine injection at a convulsive dose in rats.

## 2. Results

### 2.1. Cx mRNA Expression

**Figure 1:**
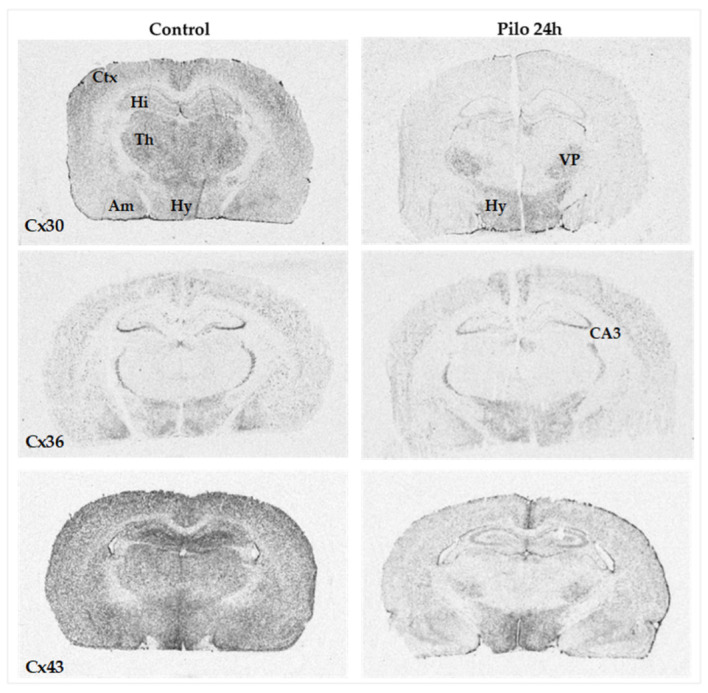
In situ hybridization analysis of *Cx30*, *Cx36,* and *Cx43* mRNAs in adult rat brains 24 h after the onset of pilocarpine-induced seizures lasting 3 h. Representative autoradiograms obtained from brain coronal sections using specific antisense probes for each connexin mRNA. Note the dramatic decrease in *Cx30* and *Cx43* mRNA levels at 24 h. Am, amygdaloid nuclei; Ctx, cerebral cortex; Hi, hippocampus; Hy, hypothalamus; Th, thalamus; VP, ventroposterior thalamic complex; CA3, CA3 subfield of the hippocampal region.

In the control rats, the distribution of *Cx* mRNAs (Figure 1) was similar to that described in previous studies [1,34].

In brief, at the cortical level, the *Cx30* transcript expression prevailed in the motor and somatosensory cortical areas, with a laminar distribution, and showed a relatively lower expression in the entorhinal and piriform cortices and hippocampus. At diencephalic levels, *Cx30* mRNA was widely and intensely expressed throughout the thalamus and hypothalamus. *Cx36* mRNA showed a more discrete, although widespread, distribution in the telencephalon and diencephalon, with prevalence in the pyramidal cell layer of the CA3/CA4 hippocampal fields and in the reticular thalamic nucleus. The *Cx36* transcript expression was also observed throughout the cerebral cortex and the hypothalamus, with a relatively low expression in the dorsal thalamus. The *Cx43* mRNA expression was relatively high throughout the brain, and widely distributed in the gray matter regions.

The three transcripts showed different responses to the ictal challenge on the first day after SE onset. With in situ hybridization, downregulation of *Cx30* and *Cx43* mRNA levels was observed in the neocortical and limbic cortical areas, including the hippocampal formation, and in the thalamus (Figure 1). Interestingly, in situ hybridization did not show a variation in *Cx43* and *Cx30* mRNAs in the hypothalamus, as well as in the ventroposterior complex of the thalamus, indicating regional selectivity (Figure 1). On the other hand, no significant changes in *Cx36* transcript expression were observed in the same animals, including the hippocampus and the reticular thalamic nucleus in which the expression persisted at relatively high levels (Figure 1).

As major changes were observed for glial connexins, we further analyzed Cx43 and Cx30 at the mRNA and protein level.

**Figure 2:**
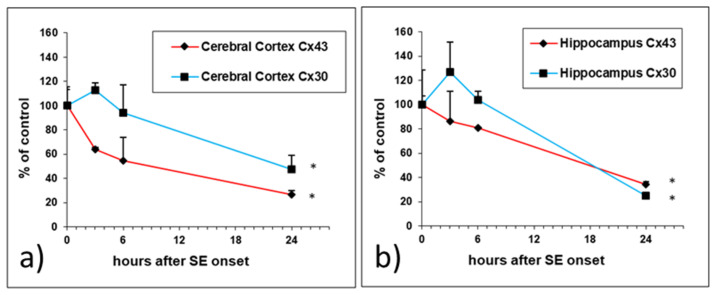
(**a**,**b**) *Cx43* and *Cx30* mRNA levels measured by quantitative real-time RT-PCR in the cerebral cortex (**a**) and hippocampus (**b**) at 3 h, 6 h, and 24 h after the onset of pilocarpine-induced seizures lasting 3 h. The results are expressed as a percentage of the control and are the mean ± SEM (*n* = 3). * *p* < 0.05 versus the control.

Quantitative analyses of the brain with real-time RT-PCR confirmed that the data observed with in situ hybridization, showing that the downregulation of *Cx30* and *Cx43* transcript levels, was significant at 24 h (Figure 2a,b).

The regional evaluation revealed that *Cx43* mRNA downregulation was already detectable after 3 h in the neocortex and hippocampus, and then progressed, reaching a significant decrease at 24 h (−74% in the neocortex and −66% in hippocampus; *p* < 0.05 versus control, Student’s t test) (Figure 2a). A biphasic variation was detected for *Cx30* mRNA levels, with a trend towards an increase at 3 h (+12% in the neocortex and +27% in hippocampus), followed by a progressive decline that was significant at 24 h (−53% in the neocortex and −75% in the hippocampus; *p* < 0.05 versus control, Student’s *t* test).

### 2.2. Cx43 and GFAP Immunofluorescence

**Figure 3:**
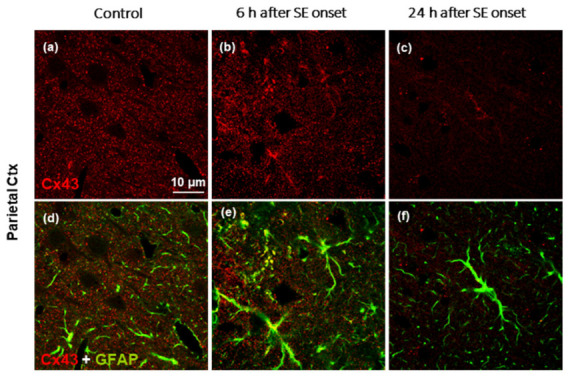
Immunolabeling of Cx43 revealed as punctate staining of the neuropil (**a**–**c**) and simultaneous visualization (merging) of Cx43 and glial fibrillary acidic protein (GFAP) immunopositivity of astrocytes (**d**–**f**) in the parietal cortex under confocal microscopy in the control animals (**a,d**), and at 6 h (**b**,**e**) and 24 h (**c**,**f**) after the onset of pilocarpine-induced seizures lasting 3 h. Note the downregulation of Cx43 at 24 h.

Cx43 immunofluorescence appeared in the control animals as fine punctate labeling, which was widely and homogeneously distributed (Figure 3a).

Cx43 immunolabeling appeared to decrease progressively after SE in the neocortex, hippocampus, and in several thalamic nuclei, with a marked decrease at 24 h (parietal cortex shown in Figure 3c). Double immunofluorescence for the simultaneous visualization of Cx43 protein immunosignal and GFAP immunopositivity of astrocytes (Figure 3d–f) revealed that the decrease in Cx43 was in contrast with GFAP immunolabeling at 24 h (Figure 3f).

### 2.3. Microglia and IL-1β Expression

Astrocytic Cx expression can be regulated by activated microglial cells via the release of proinflammatory cytokines [21,42,43]. Therefore, microglia activation and the expression of the proinflammatory cytokine interleukin (IL)-1β were also investigated after pilocarpine-induced SE.

**Figure 4:**
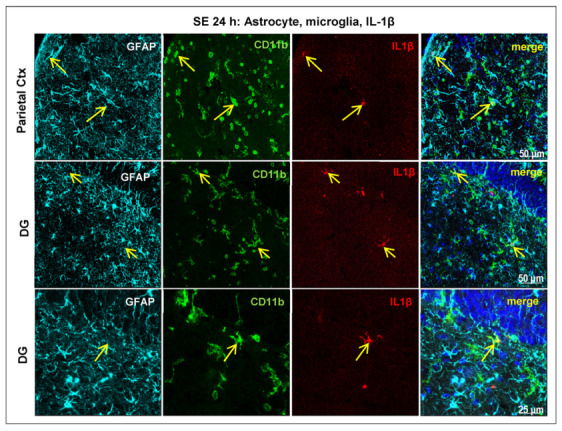
Fluorescent labeling of astrocytes (immunostaining of glial fibrillary acidic protein, GFAP), microglia (immunostaining of CD11b and IL-1β), and cellular nuclei (DAPI staining, in blue) in the parietal cortex (Ctx) and dentate gyrus (DG) 24 h after the onset of pilocarpine-induced seizures lasting 3 h. Merge images show the colocalization of IL-1β immunosignal with microglial elements (yellow arrows) in both regions, whereas no IL-1β immunopositivity is found in the astrocytes. Two different sections of DG at different magnifications are shown.

CD11b immunostaining in the brains of the control animals showed the characteristic ramified shape of resting/surveillant microglia. Changes in microglia morphology, with stouter processes, were detected 3 h after SE onset in the hippocampal CA1 field and dentate gyrus (DG) and in the superficial layers of the parietal cortex, and increased (hypertrophy and retraction of cytoplasmic processes) 6 h after SE. Activated microglial cells exhibiting a round shape and short stout processes were well evident 24 h in several brain areas, especially in the neocortex and hippocampus (Figure 4).

Few IL-1β-positive cells were detected 3 h after SE onset in the superficial layer of the parietal cortex in the hippocampus. After 24 h of SE, numerous IL-1β-ir cells were observed in the cortex (Ctx, Figure 4) in both the supra- and infragranular layers of the parietal cortex, in the cingulate cortex and hippocampus (DG, Figure 4). IL-1β immunopositivity was mainly colocalized with microglial cells and was not observed in the colocalization with astrocytes (Figure 4).

### 2.4. Neuronal Cell Death

It is well-known that pilocarpine-induced protracted seizures are followed by neuronal degeneration in several brain regions [44,45,46]. In order to investigate the regional correspondence between the observed Cx expression changes and neurodegenerative events, FJB and TUNEL positivity were analyzed in the brain sections from the control and treated animals. FJB is a sensitive and reliable marker for degenerating neurons after pilocarpine- or kainate (KA)-induced SE or other types of brain insults [47,48,49], and by the TUNEL method for the detection of apoptotic cells [1].

**Figure 5:**
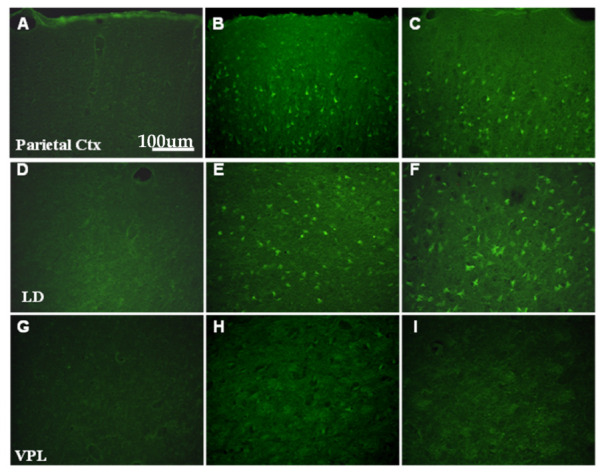
The plate illustrates FluoroJade B (FJB) staining of neurons, indicative of ongoing neuronal degeneration, 6 h (**B**,**E**,**H**) and 24 h (**C**,**F**,**I**) after the onset of pilocarpine-induced seizures lasting 3 h. No FJB-stained cells are evident in the control animals (**A**,**D**,**G**). Note the occurrence of numerous FJB-positive cells in regions such as the parietal cortex (Ctx in (**B**,**C**)) and thalamic domains (laterodorsal thalamic nucleus, LD, in (**E**,**F**)) in which the astrocytic Cx expression was seen to decrease after pilocarpine-induced status epilepticus. The thalamic ventroposterolateral nucleus (VPL in (**H**,**I**)), in which *Cx43* and *Cx30* expression did not show significant variation after SE (see Figure 1), is devoid of degenerating cells.

**Figure 6:**
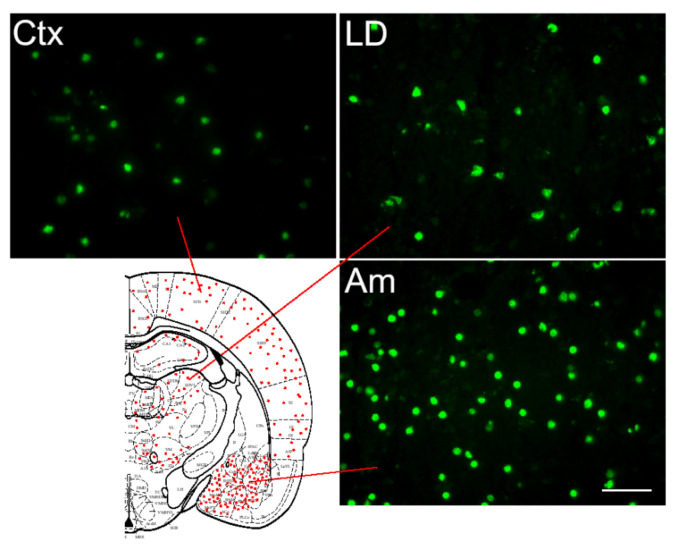
TUNEL labeling, indicative of apoptotic cells, in the cerebral cortex (Ctx), laterodorsal thalamic nucleus (LD), and amygdala (Am), and the distribution of TUNEL-positive apoptotic cells in a coronal section of rat brains (at a Bregma level −3.14 of the atlas of Paxinos and Watson (1986) [50]) 24 h after the onset of pilocarpine-induced status epilepticus.

A relatively high number of FJB-stained and TUNEL-positive cells were detected after SE in areas known to be affected by pilocarpine-induced seizures, such as Ctx, amygdala (Am), and the laterodorsal thalamic nucleus (LD) (Figure 5 and Figure 6).

In the thalamus, a discrete distribution of both FJB and TUNEL stained cells was observed (Figure 5E,F and Figure 6), with the sparing of some domains, such as the ventroposterior thalamic nuclei (Figure 5H,I and Figure 6). Thus, it is interesting to note that FJB and TUNEL-positive cells were observed in the same brain areas, such as Ctx, LD, and Am, which exhibited a marked downregulation of the expression of glial *Cx* transcripts (cf. Figure 1).

## 3. Discussion

The present study points out that, at variance with neuronal Cx36 mRNA, the expression of the *Cx30* and *Cx43* gap junctional genes expressed by astrocytes is significantly affected in the rat neocortex, hippocampus, and thalamic domains 24 h following SE onset. The event showed a regional prevalence in areas showing early and marked neurodegenerative phenomena, including apoptotic cell death, as well as microglial cell activation with the presence of amoeboid microglial cells and IL-1β expression. These sets of findings are discussed below.

### 3.1. The Acute Response of Astrocytic Cxs to Pilocarpine-Induced SE

Consistent with the present study, neuronal Cx36 expression was not found to vary after pilocarpine-induced SE in rats [35,38], although Wu et al. [36] found a decrease in mice (see Table 1). Therefore, we focused our attention mainly on astrocytic Cxs.

The data hitherto reported in the studies investigating astrocytic Cx response to pilocarpine-induced SE were obtained in the hippocampus (cf. Table 1), where the overall expression was not found to vary significantly 2 h [35,38] or 4 h [36] after SE onset in rats and mice, respectively, which is grossly consistent with the present findings. However, in contrast with the present data, the Cx43 transcript and protein expressions in the mouse hippocampus were found to be unchanged 24 h after the onset of SE lasting about 7 h [36], or to be increased 3 h and 24 h after SE lasting 1 h [37]. Although there was a difference in experimental parameters, especially concerning the duration of SE, which was here interrupted after 3 h, these discrepancies are puzzling.

Variances in the expression pattern of Cxs increase if different animal models of TLE such as kainic acid, kindling, and 4-aminopyridine are considered [1,9,16,34,51,52,53,54]. The different results reported in these studies suggest that the Cxs expression after SE may depend on the animal model, specific brain area, time point, and seizure duration [23].

Significant *Cx43* and *Cx30* transcript downregulation 24 h after SE onset was observed in the present study, not only in the hippocampus, but also in several other brain regions, with a regional selectivity. In particular, those regions corresponded in the same animals to the neocortical, limbic, and thalamic domains in which ongoing neurodegenerative phenomena were also found.

### 3.2. Neurodegeneration

The early and marked neurodegenerative phenomena in acute seizures detected in this study confirm and extend what has been described previously by some of the authors of the present work in the hippocampus and thalamus after pilocarpine-induced SE [44,46,55], as well as by other authors [45,56,57,58]. In particular, Fujikawa et al. [45] found neuronal damage occurring very early after pilocarpine injection, starting in the CA1 and CA3 hippocampal regions 20 min after SE, and involving many more hippocampal and cortices areas after 1–3 h. Jung et al. [56] reported no apparent neuronal loss in the hypothalamus, striatum, and globus pallidus after SE.

The relationship between neuronal degeneration and glial Cxs expression is still not clear.

In the cell cultures, neuronal dysfunction and death, together with brain macrophages proliferation, contribute to the downregulation of the Cx43 expression and gap junctional communication [59]. In this case, the contact between the astrocyte and brain macrophage seems to be necessary for the inhibition of the communication through gap junctions. On the other side, Bedner et al. [21] found that a decreased astrocytic coupling precedes apoptotic neuronal death in a unilateral intracortical kainate injection model of TLE.

In our study, the glial connexin expression decreased after SE in the brain regions where the neurodegenerative phenomena occurred, suggesting a correlation between the downregulation of astrocytic Cxs and neuronal cell death and/or a correlation with events that both cause neuronal cell death and affect astrocytic Cxs expression.

### 3.3. Microglia and Cytokines

Pilocarpine-induced seizures are followed by microglial cell activation with a maximal peak 24–48 h post-seizure [60]. Activated microglia has been proposed to exert an effect on the regulation of astrocytic Cx43; Meme et al. [42] reported that the activation of microglial cells in a co-culture with astrocytes produced an inhibitory effect on the astroglial Cx43 expression and suppressed the gap junctional communication between astrocytes.

The activated microglia produced cytokines, which could influence cell-to-cell communication [61]. An inhibitory effect on astrocytic gap junctional coupling exerted by proinflammatory cytokines has been observed in vitro [42,62], as well as in vivo [21] and after febrile seizures [15]. A correlation between the main distribution of IL-1β-labeled elements and the regions characterized by neuronal damage in the lithium-pilocarpine model was previously shown 12 h post-injection, persisting at 24 h, then returning to basal levels within 6 days [63].

In the present study, we show that, in acute pilocarpine-induced seizures, IL-1β is increasingly expressed by microglial cells 3 to 24 h after SE in the brain areas where both astrocytic Cxs downregulation and neurodegenerative phenomena occur in response to SE.

### 3.4. Concluding Remarks

Altogether, the present findings indicate that the expression of astrocytic *Cx43* and *Cx30* transcripts and Cx43 protein exhibit a marked downregulation at the end of the first day after SE onset, with a regional prevalence that corresponds to early neuronal damage in cortical and thalamic domains. Such a decrease may be correlated to neuronal injury through the release of chemical messengers induced by brain injury and microglia activation, such as IL-1β.

Further studies are necessary to establish if such astroglial Cxs downregulation play a protective role, decreasing the gap junction mediated transmission of “death signals”, or contribute to the neuronal damage mechanism.

## 4. Material and Methods

### 4.1. Animals, Treatment, and Experimental Design

Adult male Wistar rats (250–350 g body weight) were used in this study. All efforts were made to minimize the number of animals used and to avoid their suffering. Rats were maintained under veterinarian assistance and controlled environmental parameters, with food and water ad libitum. The induction of SE in animals was obtained by ip injection of the cholinergic muscarinic agonist pilocarpine (pilocarpine nitrate salt, P6628, Sigma-Aldrich, St. Louis, MO, USA) at a dose of 360 mg/kg. To minimize the peripheral effects, pilocarpine was preceded by 30 min by sc injection of methylscopolamine (1 mg/kg, Sigma-Aldrich). After the injection of the pilocarpine bolus, 64% of the treated rats successfully developed generalized SE and were used for further experiments. Seizure severity was scored based on the Racine scale (1972) [64], as follows: 1 = motionlessness, eye closure, ear and vibrissae twitching, sniffing, salivation, and orofacial clonus; 2 = head nodding and mastication associated with more severe orofacial clonus; 3 = unilateral forelimb clonus; 4 = rearing with bilateral clonus; and 5 = rearing and falling accompanied by generalized tonic-clonic seizures. SE onset was defined by direct observation as the recurrence of at least two seizures, either stage 4 or 5, within a time frame of 30 min. In general, stage 1 started no later than 10 min after the pilocarpine injection, followed shortly by stage 2. Stages 3 and 4 usually started within 30 min after injection, while stage 5 and generalized SE developed between 30 and 90 min following pilocarpine injection. Once initiated, SE was characterized by the occurrence of self-sustained seizures every 5–15 min, persisting until Diazepam treatment. The duration of SE was standardized by Diazepam injection (1–3 mg/kg im) 3 h after SE onset, and its termination was confirmed by visual inspection. The rats were sacrificed 3 h, 6 h, and 24 h after SE onset and the numbers of animals per time-point and per each analysis are reported in Table 2. Animals that did not respond properly to Diazepam treatment (10%) and animals dying before each established time point (33% of total) were excluded from the analysis.

The control animals received methylscopolamine, as above, followed after 30 min by ip injection of phosphate-buffered saline (PBS); they were then treated 3 h later with diazepam as above, and sacrificed 24 h after PBS injection. The brains were destined for the investigation of the expression of *Cx* transcripts, for immunohistochemical analyses, and for the study of cell death (Table 2).

Cx expression was investigated with in situ hybridization and real-time RT-PCR (Table 2). For this purpose, animals that entered SE and the control animals were sacrificed by decapitation. The brains destined for in situ hybridization were rapidly dissected out, frozen in liquid nitrogen, and kept at −80 °C until use. For regional RT-PCR investigation, the hippocampus and frontoparietal cortex were first dissected out on ice, homogenized 1:10 in a solution of guanidinium thiocyanate [65], and frozen in dry ice.

The animals destined for the immunohistochemical analyses (Table 2) were perfused transcardially, under deep anesthesia, with PBS followed by 4% paraformaldehyde in PBS. The brains were dissected out and soaked in sucrose 30% for cryoprotection.

FluoroJade B (FJB) histochemistry was used to reveal the degenerating neurons [66] in the sections from the brains of the animals destined for Cx43 immunohistochemistry. The terminal deoxynucleotidyltransferase-mediated biotinylated UTP nick end labeling (TUNEL) method was used to label the apoptotic cells in the section series adjacent to those processed for in situ hybridization.

### 4.2. In Situ Hybridization

Cryostat sections, 14 µm thick, were thawed onto 3-aminopropyl ethoxysilane-coated slides. Following fixation in 4% paraformaldehyde for 15 min, the slides were rinsed twice in PBS and once in distilled water. The tissue was deproteinated in 0.2 M HCl for 10 min, acetylated with 0.25% acetic anhydride in 0.1 M ethanolamine for 20 min, and dehydrated with increasing concentrations of ethanol. The slides were incubated for 16 h in a humidified chamber at 52 °C with 8 × 10^5^ cpm of probe in a 70 μL hybridization cocktail (50% formamide, 20 mM Tris-HCl, pH 7.6, 1 mM EDTA pH 8.0, 0.3 M NaCl, 0.1 M dithiothreitol, 0.5 g/mL yeast tRNA, 0.1 μg/mL poly-A-RNA, 1X Denhardt’s solution and 10% dextran sulfate). The slides were washed twice in 1X SSC at 62 °C for 15 min, and then in formamide-SSC (1:1) at 62 °C for 30 min. After additional washing in 1X SSC at 62 °C, single-stranded RNA was digested by RNAse treatment (10 μg/mL) for 30 min at 37 °C in 0.5 M NaCl, 20 mM Tris-HCl pH 7.5, and 2 mM EDTA. The tissue was washed twice with 1X SSC at 62 °C for 30 min before dehydration in ethanol and air drying.

For regional localization of the mRNA, hybridized sections were exposed for three weeks to beta-Max Hyperfilm (GE Healthcare, Amersham, UK) and subsequently coated with NTB-2 photoemulsion diluted 1:1 in water (Eastman-Kodak Co., Rochester, NY, USA), and stored in desiccated light-tight boxes at 4 °C for 4 weeks. The slides were then developed with D19 (Eastman-Kodak Co.), fixed with Al-4 (Agfa Gevaert, Kista, Sweden), and counterstained with cresyl violet.

Preparation and labeling of the Cx riboprobes was performed as described in previous studies [1,34]. Control of the hybridization specificity of the cRNA riboprobes was performed using sense 35S-labelled riboprobes.

### 4.3. RNA Extraction and cDNA Synthesis

The total RNA was extracted as described by Chomczynski and Sacchi [65], and 5 μg were reverse transcribed with 150 ng of random hexamers and 200 units of RNase H-reverse transcriptase (SuperScript II Invitrogen, Life Technologies, Carlsbad, CA, USA) in a reaction mixture that contained 20 mM Tris-HCl (pH 8.4 at 25 °C), 50 mM KCl, 2.5 mM MgCl_2_, 1 mM dNTP mix, 0.01 M DTT, and 40 units of the recombinant RNase inhibitor RNaseOUT (Invitrogen). The samples were incubated at 25 °C for 10 min and then at 42 °C for 50 min. The reaction was terminated by 15 min of incubation at 70 °C. After cooling the samples in ice, two units of RNase H were added, and the samples were incubated at 37 °C for 20 min.

### 4.4. Quantitative Real-Time RT-PCR for Cx Transcripts

Quantitative real-time RT-PCR experiments were performed in the ABI Prism 7700 Sequence Detection System (Applied Biosystems, Foster City, CA, USA). Three sequence-specific oligonucleotides were designed by using the “Primer Express oligo design” software (Applied Biosystems) based on the sequences of the rat *Cx30* and *Cx43*: two were the forward and reverse primers used for each PCR amplification, whereas the third sequence (TaqMan Probe, Applied Biosystems) was a fluorogenic probe labeled with a fluorescent reporter dye (6-FAM) and a quencher dye (TAMRA) attached at the 5′and 3′ends, respectively. The probe was designed to hybridize the portion of PCR product between the primers. The oligonucleotides listed in Table 3 were used.

The difference in the initial amount of cDNA between the samples was normalized in every assay by quantitation using a GAPDH housekeeping gene expression (TaqMan Rodent GAPDH Control Reagent no. 4308313, Applied Biosystems) as the internal standard. Each PCR was carried out in a 50 μL final volume with TaqMan Universal PCR Master Mix (Applied Biosystems), 900 nM primers, and 200 nM probe. Finally, 1 μL of diluted cDNA (1:4) was added to each reaction. Each sample was loaded in triplicate. Standard conditions were used for the PCR amplification (50 °C for 2 min, 95 °C for 10 min, followed by 50 cycles at 95 °C for 15 s and 60 °C for 1 min). As negative controls, reactions without added cDNA were performed (no template controls). GAPDH PCR amplifications were carried out under the same conditions for *Cx30* and *Cx43*, except for the concentrations of primers and the probe (100 and 200 nM, respectively). Relative quantification of *Cx30* and *Cx43* mRNAs was performed by the 2^−ΔΔCt^ method, as described by Livak and Schmittingen [67], using the value obtained from the RNA samples extracted from the control animals as the calibrator. Statistical analyses were performed using Student’s *t* test.

### 4.5. Immunofluorescence

Serial sections were cut through the brain at a 40 μm thickness with a freezing microtome and were collected in six adjacent series. One series was processed free-floating for double immunofluorescence to visualize Cx43 and glial fibrillary acid protein (GFAP) as a marker of astrocytes. The sections were incubated sequentially in anti-GFAP and anti-Cx43 primary antibodies (Table 4).

GFAP was revealed with biotinylated goat anti-rabbit secondary antibody and Fluorescein avidin D; Cy^3^ donkey anti-mouse secondary antibodies (1:200) were used for the Cx43. The material was examined using a Zeiss LSM 510 confocal microscope equipped with argon (488 nm) and helium/neon (543 nm) excitation beams.

An additional series of sections was processed free-floating for triple immunofluorescence to visualize GFAP, CD11b as a marker for the microglia, and the proinflammatory cytokine IL-1β. The sections were first incubated in 5% normal donkey serum (NDS) and 0.3% Triton-X-100 in PBS, and then incubated overnight in PBS containing 1% NDS and anti-GFAP, anti-CD11b, and anti-IL-1β primary antibodies (Table 4). After washing, the sections were incubated for 2 h in a solution containing 1% NDS and the secondary antibodies made in donkey: anti-rabbit IgG Alexa647, anti-mouse Alexa488, and anti-goat Alexa546, all diluted 1:1000 and purchased from Invitrogen (Thermo Fisher Scientific, Waltham, MA). The sections were then counterstained with the fluorescent nuclear marker 4′,6-diamidino-2-phenylindole (DAPI), mounted with an anti-fading glycerol-based medium containing 0.1% paraphenylendiamine, and coverslipped. The material was examined using a Leica SP5 confocal microscope (Leica, Manheim, Germany).

### 4.6. Fluoro-Jade B and TUNEL Staining

FJB histochemistry was performed in series of sections from the brains destined for the immunohistochemical analyses. Sections from perfused rats, mounted on gelatinized slides, were immersed for 5 min in a solution of 1% sodium hydroxide in 80% ethanol, and then for 2 min in 70% ethanol. After rinsing in distilled water, the sections were transferred to a solution of 0.06% potassium permanganate for 10 min, rinsed again, and stained for 20 min in a solution prepared from a 0.01% stock solution of FJB (Histo-Chem, Jefferson, AR) in 0.1% acetic acid. Finally, they were washed and analyzed under a fluorescence microscope.

The presence of apoptotic cells was evaluated by the TUNEL method in a series of sections from the brains destined for in situ hybridization (Table 2). Frozen sections were fixed for 10 min with acetone–methanol (1:1) at −20 °C, and permeabilized with 0.1% Tween−20/1% bovine serum albumin (BSA)/PBS. Fluorescein-labelled UTP was added together with TdT (Roche, Mannheim, Germany) and the sections were incubated for 1 h at 37 °C, washed, and analyzed under a fluorescence microscope.

## Figures and Tables

**Table 1:** Cx43 and Cx36 in the pilocarpine model of TLE.

Cx43 and Cx36 in the Pilocarpine Model of TLE							
Authors Year, Journal	Species	Method	Stage Post-SE		Brain Area	Cxs	
						Cx43	Cx36
Kinjo et al., 2014, PLoS One [35]	rat	*RT-PCR,* *WB, Immuno*	**Acute**	2 h	**Hipp, radiatum, pyr**	2 h = (radiatum↓, pyr ↑)	=
			**Silent**	3 d	**Hipp, radiatum, pyr**	↑	=
Wu et al., 2015, Exp. Brain Res. [36]	mouse	*RT-PCR,* *WB,* *Immuno*	**Acute**	4 h, 24 h	**Hipp, CA1-CA3, DG gran**	=	
			**Silent**	1 w	**Hipp, CA1-CA3, DG gran**	↑	
			**Chronic**	2 m	**Hipp, CA1-CA3, DG gran**	↑	
Motaghi et al., 2017, Iranian Biom. J. [38]	rat	*WB*	**Acute**	2 h	**Hipp**	=	=
			**Silent**	72 h	**Hipp**	↑	=
			**Chronic**	1 w	**Hipp**	=	=
Wu et al., 2018, Epilepsy Res. [39]	mouse	*RT-PCR,* *WB,* *Immuno*	**Acute**	1 h, 4 h	**Hipp**		↓
			**Silent**	1 w	**Hipp**		↓
			**Chronic**	2 m	**Hipp**		↓
Ran et al., 2018, Epilepsy Res. [40]	mouse	*WB*	**Silent**	3 d	**Hipp**	↑	=
Men et al., 2019, Brain Res. Bull. [37]	mouse	*WB*	**Acute**	3 h, 24 h	**Hipp**	↑	
			**Silent**	7 d	**Hipp**	↑	
			**Chronic**	15 d, 30 d	**Hipp**	15 d ↑, 30 d =	

**Table 2:** Experimental procedures.

Method	Animals			
	Control	3 h after SE Onset	6 h after SE Onset	24 h after SE Onset
**ISH/TUNEL**	*n* = 2	*n* = 3	*n* = 3	*n* = 3
**RT-PCR**	*n* = 3	*n* = 3	*n* = 3	*n* = 3
**ICC/FJB**	*n* = 4	*n* = 4	*n* = 4	*n* = 4

FJB, FluoroJade B; ICC, immunocytochemistry; ISH, in situ hybridization; m, month; SE, status epilepticus; TUNEL, terminal deoxynucleotidyltransferase-mediated biotinylated UTP nick end labeling.

**Table 3:** PCR primers used in the gene expression analysis.

Gene mRNA	Primer Sequence (5′-3′)	
r*Cx30*	forward primer	5′-AGGAGGGATTTTGCAGTGGTT-3′
	reverse primer	5′-GCGCACGCTCCTGAGTCT-3′
	fluorogenic probe	5′-FAM-TTGGACTGGACGACGCACTGGAAGT-TAMRA-3′
r*Cx43*	forward primer	5′-CGGCTTCACTTTCATTAAGTGAAAG-3′
	reverse primer	5′-TAGGCTTGGACCTTGTCCAGAA-3′
	fluorogenic probe	5′-FAM-ACATGGGTGACTGGAGT-TAMRA-3′

**Table 4:** Antibodies used in this study.

Antigen	Host	Immunogen	Supplier	Catalog#	Dilution
GFAP	Rabbit	Purified Bovine GFAP	DAKO (Agilent, Santa Clara, CA, USA)	Z0334	1:500
CX43	Mouse	Synthetic connexin-43 peptide (362–381)	Sigma (Sigma-Aldrich, Milan, Italy)	C8093	1:3000
CD11b	Mouse	Resident rat peritoneal macrophages	Bio-Rad (formely Serotec) (Oxford, UK)	MCA275	1:500
IL-1β	Goat	Epitope mapping at the C-terminus of IL-1β of rat origin	Santa Cruz Biotec. (Dallas, TX, USA)	Sc-1252	1:400
